# Increased mortality associated with methicillin-resistant *Staphylococcus aureus *infection in the ICU: results from the EPIC II study

**DOI:** 10.1186/cc9654

**Published:** 2011-03-11

**Authors:** H Hanberger, S Walther

**Affiliations:** 1 Clinical and Experimental Medicine, Linköping, Sweden

## Introduction

Controversy continues regarding whether methicillin resistance increases mortality risk in *Staphylococcus aureus *infections. We assessed the role of methicillin resistance on survival of patients in the EPIC II study cohort with *S. aureus *infection.

## Methods

The EPIC II point-prevalence study of infection in critically ill patients was performed on 8 May, 2007. Demographic, physiological, bacteriological and therapeutic data were collected for all adult patients in 1,265 participating ICUs from 75 countries on the study day. ICU and hospital outcomes were recorded. We compared characteristics of patients with methicillin-sensitive (MSSA) and methicillin-resistant (MRSA) *S. aureus *infection. Co-morbidities, age, simplified acute physiology system (SAPS) II score, site of infection, geographical region, and MRSA/MSSA were entered into a multivariable model and adjusted odds ratios (ORs) (95% CI) were calculated for ICU and hospital mortality rates.

## Results

On the study day, 7,087 of the 13,796 patients (51%) were classified as infected. There were 494 patients with MRSA and 505 patients with MSSA infections. There were no significant differences between the two groups in use of mechanical ventilation or hemofiltration/hemodialysis. Cancer and chronic renal failure were more prevalent in MRSA than in MSSA patients. ICU mortality rates were 29.1% and 20.5%, respectively (*P *< 0.01) and corresponding hospital mortality rates were 36.4% and 27.0% (*P *< 0.01). Multivariable analysis of hospital mortality for MRSA infection showed an adjusted OR of 1.48 (1.05 to 2.10), *P *= 0.03.

## Conclusion

In ICU patients, MRSA infection is more common in patients with co-morbid conditions, such as cancer and chronic renal failure, and is independently associated with an almost 50% higher odds of hospital death compared with MSSA infection.
